# Apolipoprotein A4 Elevates Sympathetic Activity and Thermogenesis in Male Mice

**DOI:** 10.3390/nu15112486

**Published:** 2023-05-26

**Authors:** Hsuan-Chih N. Kuo, Zachary LaRussa, Flora Mengyang Xu, Kathryn West, Leslie Consitt, William Sean Davidson, Min Liu, Karen T. Coschigano, Haifei Shi, Chunmin C. Lo

**Affiliations:** 1 Department of Biomedical Sciences, Heritage College of Osteopathic Medicine and Diabetes Institute, Ohio University, Athens, OH 45701, USA; nico0317002@gmail.com (H.-C.N.K.); zl928415@ohio.edu (Z.L.); kt070217@ohio.edu (K.W.); consitt@ohio.edu (L.C.); coschigk@ohio.edu (K.T.C.); 2 Department of Biological Sciences, Ohio University, Athens, OH 45701, USA; 3 Department of Biology, Miami University, Oxford, OH 45056, USA; xum3@miamioh.edu (F.M.X.); shih@miamioh.edu (H.S.); 4 Department of Pathology and Laboratory Medicine, University of Cincinnati, Cincinnati, OH 45237, USA; davidswm@ucmail.uc.edu (W.S.D.); lium@ucmail.uc.edu (M.L.)

**Keywords:** thermogenesis, plasma lipids, brown adipose tissue, liver, caloric intake, glucose tolerance

## Abstract

Long-chain fatty acids induce apolipoprotein A4 (APOA4) production in the small intestine and activate brown adipose tissue (BAT) thermogenesis. The increase in BAT thermogenesis enhances triglyceride clearance and insulin sensitivity. Acute administration of recombinant APOA4 protein elevates BAT thermogenesis in chow-fed mice. However, the physiological role of continuous infusion of recombinant APOA4 protein in regulating sympathetic activity, thermogenesis, and lipid and glucose metabolism in low-fat-diet (LFD)-fed mice remained elusive. The hypothesis of this study was that continuous infusion of mouse APOA4 protein would increase sympathetic activity and thermogenesis in BAT and subcutaneous inguinal white adipose tissue (IWAT), attenuate plasma lipid levels, and improve glucose tolerance. To test this hypothesis, sympathetic activity, BAT temperature, energy expenditure, body weight, fat mass, caloric intake, glucose tolerance, and levels of BAT and IWAT thermogenic and lipolytic proteins, plasma lipids, and markers of fatty acid oxidation in the liver in mice with APOA4 or saline treatment were measured. Plasma APOA4 levels were elevated, BAT temperature and thermogenesis were upregulated, and plasma triglyceride (TG) levels were reduced, while body weight, fat mass, caloric intake, energy expenditure, and plasma cholesterol and leptin levels were comparable between APOA4- and saline-treated mice. Additionally, APOA4 infusion stimulated sympathetic activity in BAT and liver but not in IWAT. APOA4-treated mice had greater fatty acid oxidation but less TG content in the liver than saline-treated mice had. Plasma insulin in APOA4-treated mice was lower than that in saline-treated mice after a glucose challenge. In conclusion, continuous infusion of mouse APOA4 protein stimulated sympathetic activity in BAT and the liver, elevated BAT thermogenesis and hepatic fatty acid oxidation, and consequently attenuated levels of plasma and hepatic TG and plasma insulin without altering caloric intake, body weight gain and fat mass.

## 1. Introduction

Dietary lipids elevate BAT thermogenesis through the activation of vagus and sympathetic nerves in animals and humans to reduce energy surplus [1,2,3,4]. In BAT, sympathetic stimulation leads to an increase in adipose triglyceride lipase (ATGL) for increased intracellular TG hydrolysis and free fatty acids derived from intracellular lipolysis for fatty acid beta oxidation, where uncoupling proteins (UCP) are involved in heat production [5,6]. Stimulation of BAT thermogenesis enhances energy expenditure, TG clearance, and insulin sensitivity and reduces fat mass and body weight gain [7,8,9]. In contrast, reduced BAT activity and thermogenesis, due to lower lipid-induced sympathetic activity, have been found in obese humans and animals [10,11,12]. Thus, a better understanding of the factors for the elevation of BAT thermogenesis could have health benefits in combating obesity-related complications.

APOA4 is synthesized in the enterocytes of the small intestine in response to dietary lipids [13]. APOA4 production is induced by long-chain fatty acids but not short- or medium-chain fatty acids [14]. Additionally, increased doses of long-chain fatty acids elevate APOA4 synthesis and secretion [15]. After the absorption of fatty acids by the intestinal enterocytes, the long-chain fatty acids are packaged into chylomicrons, whereas APOA4 is coated on the surface [13,16]. APOA4 production is chylomicron-dependent, and subsequently chylomicrons with APOA4 are released into the lymph and eventually into the circulation [17]. The small intestine contributes approximately 60% of the plasma APOA4 pool [13]. In the circulation, most of the APOA4 is lipoprotein-free, and some APOA4 is transferred from chylomicrons to high-density lipoproteins [13]. 

APOA4 regulates BAT thermogenesis, lipid transport, glucose metabolism, cholesterol efflux, inflammation, and thrombosis, and protects against the development of atherosclerosis and obesity [13,18,19,20,21]. APOA4 also serves as a short-term satiating protein that reduces meal size and increases meal frequency but does not alter daily caloric intake [22,23]. In the presence of dietary lipids, reduced norepinephrine (NE) synthesis, impaired UCP1-dependent BAT thermogenesis, and decreased energy expenditure are found in mice with a global knockout of APOA4 (APOA4-KO mice) [24]. In chow-fed wildtype mice, acute intraperitoneal administration of APOA4 protein elevates intracellular lipolysis and thermogenesis through sympathetic activation of BAT [20]. Furthermore, APOA4 administration increases insulin secretion and improves glucose homeostasis [21]. The findings suggest that peripheral effects of APOA4 enhance UCP1-dependent thermogenesis and sympathetic activity in the BAT and improve glucose homeostasis. Whether or not a continuous infusion of recombinant APOA4 protein can elevate BAT thermogenesis and sympathetic activity, attenuate plasma lipid levels and improve glucose tolerance in lean mice remains unknown. This study hypothesized that continuous infusion of mouse APOA4 protein would stimulate sympathetic activity, increase BAT thermogenesis, and consequently reduce plasma lipid levels and improve glucose homeostasis in lean mice. In the present studies, BAT temperature, BAT thermogenic and lipolytic protein levels, body weight, feeding behavior, plasma parameters, and glucose tolerance in APOA4- and saline-treated mice were investigated. 

## 2. Materials and Methods

### 2.1. Animals

Male C57BL/6J mice (n = 40 mice per group) at 10 weeks of age from Jackson Laboratory (Bar Harbor, ME, USA) were maintained in an AAALAC-accredited facility under conditions of a controlled-illumination (12:12 h) light–dark cycle, with lights on from 6:00 to 18:00 h) and controlled room temperature (28 °C). All animals had free access to water and a low-fat diet (LFD, 5% lard fat by weight, 75% carbohydrates by weight, and 20% protein by weight; # D18120703, Research Diets, Inc., New Brunswick, NJ, USA) for 10 weeks. Body weight and caloric intake were recorded weekly with a top loading balance (±0.01 g, Fisher Science Education Portable balance, SLF152, Waltham, MA, USA). All animal protocols were approved by the Institutional Animal Care and Use Committee at Ohio University.

Prior to beginning LFD feeding, the average initial body weight of the mice was 24.8 ± 1.67 g while kept on a chow diet. After the 6-week feeding of the LFD, mice were divided into saline or APOA4 treatment groups, matching body weight between groups, and implanted with an ALZET^®^ minipump that resulted in an intraperitoneal infusion of either saline or APOA4 in saline at 0.11 µL per hour (1.2 mg APOA4/kg per day) for 4 weeks while remaining on the LFD. At the end of the experiment, body composition was monitored using an LF-50 body composition analyzer (Bruker, Billerica, MA, USA) and body weight was measured the same day. After a 5 h fast, plasma, interscapular BAT, subcutaneous inguinal white adipose tissue (IWAT), and livers were collected and stored at −80 °C for further determinations.

### 2.2. Recombinant APOA4 Protein

Recombinant mouse APOA4 protein was generated using a bacterial expression system and purified according to our established protocol [25]. Briefly, APOA4 was expressed in a pET expression system and isolated from a sonicated cell extract using a nickel-chelating column. After the His-tag in the APOA4 protein was removed using the IgA protease, the mature protein was purified from the cleaved tag by a second passage through the nickel-chelating column. The recombinant APOA4 protein had previously been confirmed to be of a similar molecular mass to that of APOA4 isolated from plasma using a SDS gel. Gene sequencing and peptide analysis of the final APOA4 product via mass spectrometry were performed for verifying the sequence of the recombinant APOA4. The recombinant APOA4 protein inhibits food intake and does not cause conditioned taste aversion [25,26]. Intraperitoneal administration of recombinant APOA4 protein showed that it enters the lymph and blood within 30 min [27]. 

### 2.3. Sympathetic Activity

Sympathetic activity was assessed using the NE turnover rate according to our published protocol [20,28]. Briefly, 5 h fasted mice received an intraperitoneal injection of either saline as a vehicle or a tyrosine hydroxylase inhibitor, α-methyl-paratyrosine (Sigma-Aldrich, St. Louis, MO, USA; 250 mg/kg). After 2 h, mice received a second injection of either the vehicle or supplemental α-methyl-paratyrosine (125 mg/kg). BAT, IWAT and livers were rapidly collected and snap-frozen 4 h post-injection of the initial α-methyl-paratyrosine. All tissues were stored at −80 °C until catecholamine was measured for NE turnover rate calculation. For catecholamine measurements, tissues were homogenized in a perchloric acid-ascorbic acid solution containing the internal standard, dihydroxybenzylamide, catecholamine was extracted from the homogenate, and NE content was determined using a reverse-phase high-performance liquid chromatography (HPLC) system with electrochemical detection (Thermo UltiMate^TM^ 3000 UHPLC system, Waltham, MA, USA) [28]. NE turnover rate was calculated using the following formula: k = (lg[NE]_0_ − lg[NE]_4_)/(0.434 × 4) and K = k[NE]_0_, where k is the rate of NE efflux, [NE]_0_ is the NE concentration of the group without the α-methyl-paratyrosine injection, [NE]_4_ is the NE concentration of the group with the α-methyl-paratyrosine injection, and K is the NE turnover rate [29].

### 2.4. BAT Temperature

Infrared thermography, a noninvasive method, is used to measure the skin temperature of interscapular BAT areas for the detection of BAT temperatures [30]. BAT temperature in LFD-fed mice was monitored before and after infusion of APOA4 or saline. The hair in the BAT region of mice was removed by shaving it one week before the determination of BAT temperature. After a 5 h fast, each mouse was transferred into a small box 15 min prior to temperature determination. BAT temperature was monitored using an infrared camera with a high-resolution heat map image (FLIR T530 sc w/24° Lens, 320 × 240, −20 °C to 650 °C, FLIR, Nashua, NH, USA) [31,32]. Three BAT thermograms from each mouse were collected, and the BAT thermograms were analyzed with the FLIR Research IR Max 4 software for the quantification of BAT temperature. BAT temperature, Δ, represented the difference between before and after the APOA4 or saline treatments. 

### 2.5. Thermogenic Protein and Plasma Apolipoprotein 

BAT, IWAT and liver proteins were extracted with a radioimmunoprecipitation assay (RIPA) lysis buffer system (Santa Cruz Biotechnology, Dallas, TX, USA). For the process of immunoprecipitation, amounts 10 µL of extracted BAT, IWAT and liver proteins were incubated with 5 µL of undiluted UCP1 antibody (#50-173-4107, Proteintech, Rosemont, IL, USA), ATGL antibody (#2439S, Cell Signaling Technology, Inc., Danvers, MA, USA), cluster of differentiation 36 (CD36) antibody (#ab124515, Abcam, Waltham, MA, USA) or beta-tubulin antibody (#2128L, Cell Signaling Technology, Inc.) and Dynabeads Protein A using a Dynabeads Protein A immunoprecipitation kit (Invitrogen, Vilnius, Lithuania) overnight at 4 °C. The eluted sample (30 µL) or plasma (2 µL) was loaded onto a 4–20% Tris-HCl gradient gel (Bio-Rad Laboratories, Hercules, CA, USA) for the measurement of UCP1, ATGL, and beta-tubulin (BAT and IWAT); CD36, carnitine palmitoyltransferase 1 (CPT1), carnitine palmitoyltransferase 2 (CPT2), and beta-actin proteins (liver); or APOA4 and APOA1 proteins (plasma). The sodium dodecyl sulfate (SDS)-polyacrylamide gels were run at 80 volts for 1 h and at 100 volts until the protein standards were well separated, and then the proteins were transferred to a polyvinylidene difluoride membrane (Bio-Rad Laboratories). The membrane was incubated in a 5% blotting-grade blocker solution (Bio-Rad Laboratories) and then incubated with one of the following antibodies diluted in 5% bovine serum albumin: beta-tubulin (1:1000), UCP1 (1:1000), ATGL (1:1000), CD36 (1:1000), CPT1 (#15184-1-AP, 1:5000, Proteintech Group, Inc., Rosemont, IL, USA), CPT2 (#26555-1-AP, 1:1000, Proteintech group, Inc.), beta-actin (#66009-lg, 1:10,000, Proteintech Group, Inc.), polyclonal APOA4 (#PA5-14554, 1:1000, Invitrogen, Waltham, MA, USA) or APOA1 (#PA5-29557, 1:1000, Invitrogen) antibody. After incubation with the primary antibody overnight at 4 °C, the immunoblots were washed and then incubated with horseradish peroxidase (HRP)-conjugated secondary antibody (#p044901-2, 1:6000, Dako Cytomation, Santa Clara, CA, USA) for 1 h at 25 °C. Detection of the proteins involved visualizing them with the enhanced chemiluminescence system (Immobilon western chemiluminescent HRP substrate, EMD Millipore Corporation, Billerica, MA, USA) and a C-Digit blot scanner (Li-Cor Biosciences, Lincoln, NE, USA) according to our published protocols [20,23,24]. For tissue proteins, the average band intensity of each protein was measured. Levels of BAT and IWAT proteins were normalized to those of the beta-tubulin protein. Levels of liver proteins were normalized to those of the beta-actin protein. For plasma apolipoproteins, the average band intensity of APOA1 or APOA4 in saline-treated mice was considered to be 100%.

### 2.6. Energy Expenditure, Respiratory Exchange Ratio (RER) and Locomotor Activity 

APOA4- and saline-treated mice at an age of 19 weeks during the 10th week of LFD feeding (n = 10 mice per group) were acclimatized to individual metabolic cages of a comprehensive lab animal monitoring system (Columbus Instrument, Columbus, OH, USA) for 3 days. Data on energy expenditure, RER and locomotor activity were collected in 16 min intervals for 2 days. Energy expenditure was covaried for lean body mass.

### 2.7. Intraperitoneal Glucose Tolerance Test 

Five-hour-fasted mice with APOA4 or saline infusion (n = 9 or 10 mice per group) received intraperitoneal injections of 2 mg of glucose/g body weight (Sigma, St. Louis, MO, USA) in saline according to our published protocol [33]. Tail blood glucose and plasma insulin were measured before glucose administration and at 15, 30, 60 and 120 min post-injection of the glucose solution. Whole blood glucose was determined using a Bayer Contour glucometer and glucose strips (Ascensia Diabetes Care US Inc., Parsippany, NJ, USA), and plasma insulin was determined using an enzyme-linked immunosorbent assay (ELISA; see details in Section 2.8).

### 2.8. Plasma Parameters

Levels of insulin and leptin in 5 h fasted plasma were determined with commercial mouse insulin or leptin ELISA kits (#90080 or #90030, Crystal Chem, Elk Grove Village, IL, USA). Briefly, plasma (10 µL) was incubated with pre-coated anti-insulin or anti-leptin monoclonal antibodies on a microtiter plate, and the detection antibody was added. After incubation, absorbance was measured with a microplate reader (Synergy HT, BioTek Instruments Inc., Richmond, VA, USA), and the final concentrations were calculated based on the standard curve according to the manufacture’s protocol. Levels of plasma triglycerides and cholesterol were determined with Infinity triglyceride and cholesterol kits (Thermo Scientific, Middletown, VA, USA).

### 2.9. Statistical Analysis

All values are presented as mean ± SD. All data were checked for normality using the Shapiro–Wilk test. For measurements of RER, locomotor activity and plasma parameters for the intraperitoneal glucose tolerance test, repeated measures analysis of variance (ANOVA), followed by Sidak’s multiple comparisons test, was performed using GraphPad™ Prism (version 9.0, San Diego, CA, USA). Analysis of covariance (ANCOVA) for energy expenditure measurements was performed with lean mass as the covariate using SPSS version 28.0 software (SPSS Inc., Chicago, IL, USA) [34,35]. For all other data, differences between saline and APOA4 groups were analyzed using either an unpaired t-test (normally distributed data) or Mann–Whitney U test (non-normally distributed data), as mentioned in the figure legends, using GraphPad™ Prism (version 9.0) for end-point measurements. Differences were considered significant if the *p* value was <0.05. 

## 3. Results

### 3.1. Infusion of APOA4 Increases Thermogenesis and Sympathetic Activity in BAT

Intraperitoneal administration of APOA4 protein at a physiological dose of 0.6 mg/kg in a pilot study and previous report [20] elevated BAT UCP1 in chow-fed mice relative to saline treatment (*p* < 0.05, Supplementary Figure S1A). In contrast, APOA4 administration at 0.4 mg/kg or lower doses did not increase BAT UCP1 compared to saline treatment (*p* < 0.05, Supplementary Figure S1A). In addition, daily administration of APOA4 at 0.6 mg/kg twice a day for 3 weeks could elevate BAT UCP1 proteins in mice fed a chow diet at 21 °C (*p* < 0.05, Supplementary Figure S1B). The findings suggest that an acute injection of APOA4 at 0.6 mg/kg or daily administration of APOA4 at 0.6 mg/kg twice a day (i.e., 1.2 mg/kg total each day) for 3 weeks elevates UCP1-dependent BAT thermogenesis. 

**Figure 1:**
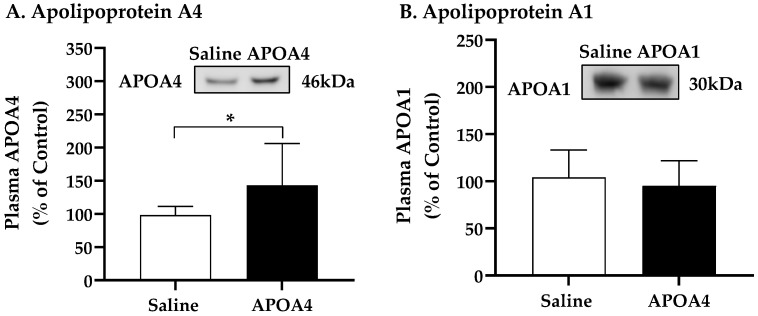
Levels of plasma apolipoprotein A4 (**A**) and A1 (**B**) in 5 h fasted mice after 4-week infusion of saline or APOA4. Plasma APOA4 or APOA1 in saline-treated mice was considered as 100%. Data are expressed as mean ± SD for 8–10 animals per group. Nonparametric data were analyzed by Mann–Whitney U test. Values with asterisks (*) represent significant differences relative to the saline-treated mice (*p* < 0.05).

**Figure 2:**
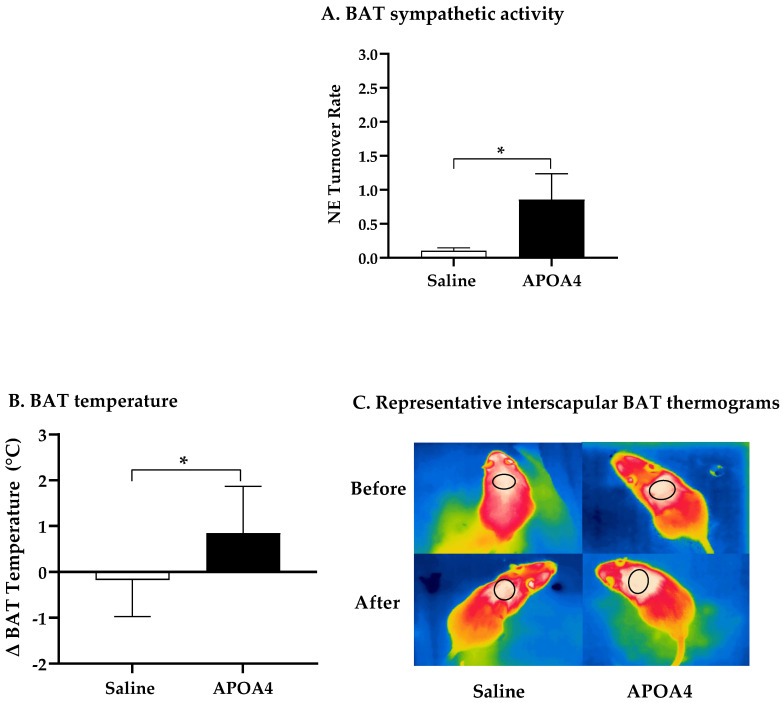

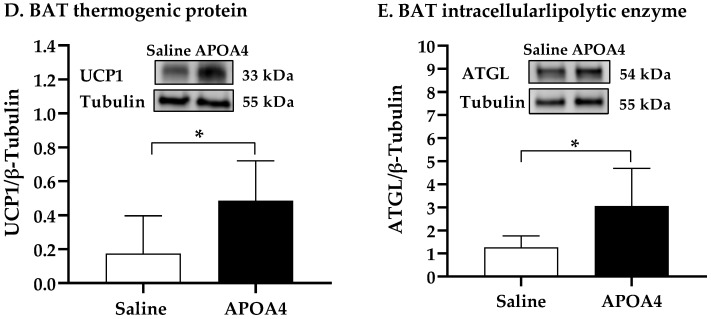
BAT NE turnover rate (**A**), BAT temperature (**B**), representative BAT thermograms (**C**), and protein levels of uncoupling protein 1 (UCP1) (**D**) and adipose triglyceride lipase (ATGL) (**E**) in BAT of mice after 4-week infusion of saline or APOA4. Infrared thermograms with circled areas were measured for interscapular BAT temperature. Data are expressed as mean ± SD for 6–10 animals per group. Data were analyzed using parametric unpaired *t*-test. Values with asterisks (*) represent significant differences relative to saline-treated group (*p* < 0.05).

Further, we investigated if the continuous infusion of APOA4 at 1.2 mg/kg a day for 4 weeks would elevate BAT thermogenesis in lean mice housed at 28 °C. In mice, continuous infusion of APOA4 for 4 weeks significantly raised plasma APOA4 levels, making them 40% higher than those in saline-treated mice (*p* < 0.05, Figure 1A). In contrast, plasma APOA1 protein levels were similar between APOA4- and saline-treated mice (Figure 1B). Because acute administration of APOA4 stimulates sympathetic activity assessed by the NE turnover rate and thermogenesis in the BAT of chow-fed mice [20], sympathetic activity and UCP1-dependent thermogenesis in the BAT of mice with a continuous infusion of either APOA4 or saline were measured. In the current experiments, APOA4 infusion significantly elevated sympathetic activity and increased BAT temperature relative to saline treatment (*p* < 0.05, Figure 2A–C). APOA4 infusion also increased levels of UCP1 protein and ATGL protein, an intracellularlipolytic enzyme [5], in BAT compared to saline treatment (*p* < 0.05, Figure 2D,E). 

**Figure 3:**
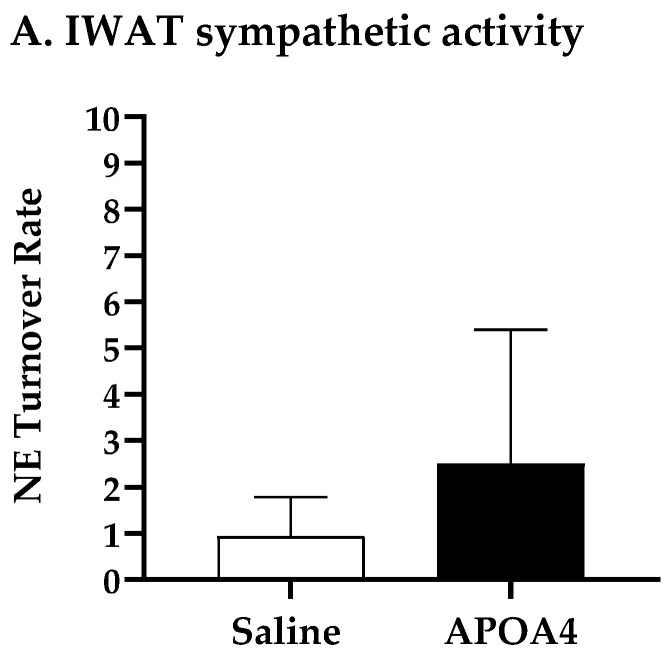

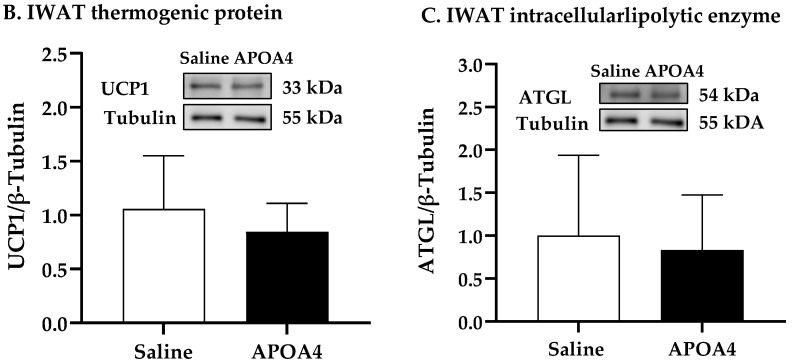
IWAT NE turnover rate (**A**), and protein levels of uncoupling protein 1 (UCP1) (**B**) and adipose triglyceride lipase (ATGL) (**C**) in IWAT of mice after 4-week infusion of saline or APOA4. Data are expressed as mean ± SD for 6–10 animals per group. Data were analyzed using parametric unpaired *t*-test. No significant differences were observed.

Sympathetic neural activation elevates UCP1-positive cells in subcutaneous IWAT that contains brown adipocytes for the induction of thermogenesis [36]. To examine if the continuous infusion of APOA4 would elevate UCP1-dependent thermogenesis in IWAT, sympathetic activity and thermogenesis in subcutaneous IWAT were assessed. APOA4 infusion did not change sympathetic activity in subcutaneous IWAT relative to saline treatment (Figure 3A). Additionally, the levels of UCP1 and ATGL in the subcutaneous IWAT of APOA4-treated mice were comparable to those in saline-treated mice (Figure 3B,C). The findings suggest that continuous infusion of APOA4 increases plasma APOA4 levels and sympathetic activity, and elevates ATGL intracellular lipolytic enzyme and UCP1-dependent thermogenesis in BAT while APOA4 infusion does not alter sympathetic activity, ATGL intralipolytic enzyme levels, or UCP1-dependent thermogenesis in subcutaneous IWAT.

### 3.2. Infusion of APOA4 Regulates Energy Homeostasis and Lipid Metabolism

**Figure 4:**
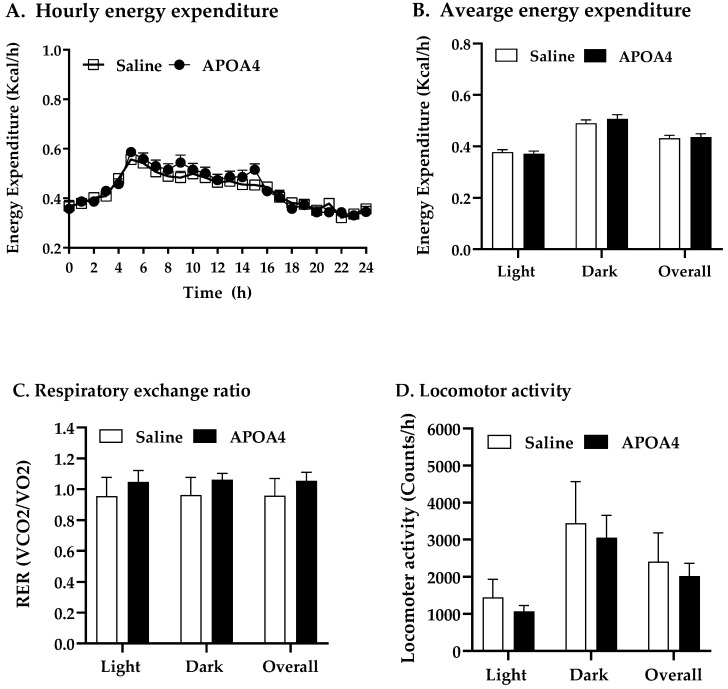
Hourly energy expenditure (**A**), average energy expenditure (**B**), average respiratory exchange ratio (RER, VCO2/VO2) (**C**), and average locomotor activity (**D**) in mice during light and dark phases of the 10th week of LFD feeding at 28 °C. Energy expenditure was covaried for lean body mass. Data are expressed as mean ± SD. n = 10 mice per group. No significant differences were observed.

APOA4 infusion increased BAT but not IWAT thermogenesis in the current study. To examine if APOA4 infusion would regulate whole-body energy homeostasis, energy expenditure, RER, and locomotor activity in saline- or APOA4-treated mice were monitored during the feeding of the LFD. APOA4 infusion did not alter lean mass or fat mass normalized by body weight compared to those saline-treated mice (Table 1). APOA4-treated mice had comparable lean body masses not normalized by body weight (18.5 ± 0.45 g) to saline-treated mice (18.4 ± 0.31 g). No significant difference in adjusted hourly energy expenditure during LFD feeding, average energy expenditure in light or dark phases or overall were observed between APOA4 infusion and saline treatment (Figure 4A,B). Additionally, the RERs in these APOA4-treated mice were comparable to their controls (Figure 4C), indicating that no significant differences in energy substrates occurred for heat production. Locomotor activity, daily caloric intake, and body weight were similar between the APOA4- and saline-treated mice (Figure 4D, Table 1). These findings suggest that continuous infusion of APOA4 does not elevate whole-body energy expenditure, locomotor activity, caloric intake, body weight or body composition in LFD-fed mice although it upregulates BAT thermogenesis.

Because the activation of BAT thermogenesis induces TG clearance and fatty acid oxidation in humans and animals [8,37] and APOA4-induced fatty acid uptake by adipose tissues in chow-fed mice [20], we investigated if APOA4 infusion would reduce plasma lipids in LFD-fed mice. Plasma levels of TG in APOA4-treated mice were lower than those in saline-treated mice (*p* < 0.05; Table 1). No significant difference in the plasma levels of cholesterol, leptin and insulin were observed between APOA4 and saline treatments (Table 1). Additionally, caloric intake was similar between these two treatments (Table 1).

### 3.3. Infusion of APOA4 Regulates Hepatic Lipid Content and Glucose Homeostasis 

**Figure 5:**
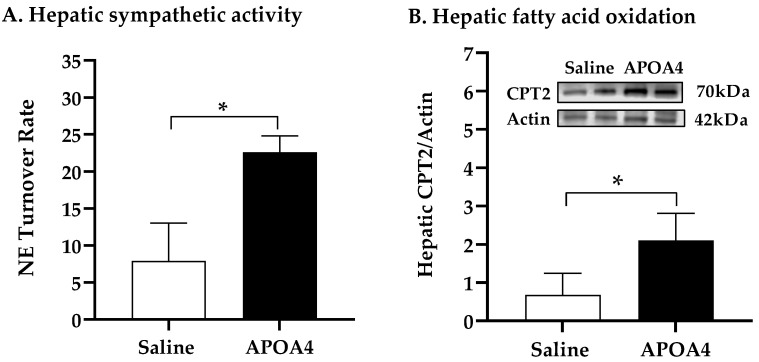

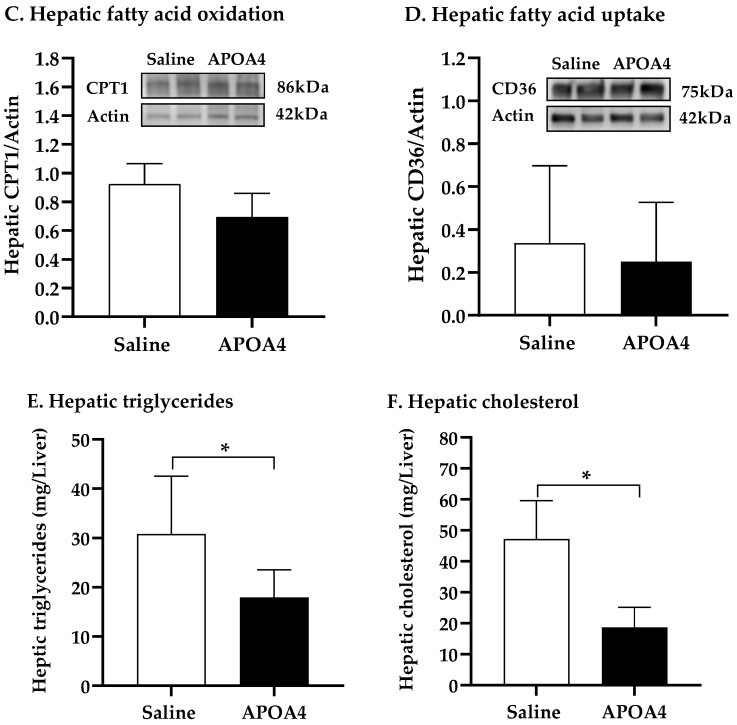
Hepatic NE turnover rate (**A**), protein levels of carnitine palmitoyltransferase 2 (CPT2) (**B**), carnitine palmitoyltransferase 1 (CPT1) (**C**), and cluster of differentiation 36 (CD36) (**D**), and amounts of triglycerides (**E**) and cholesterol (**F**) in the liver of 5 h fasted mice after 4 week infusion of saline or APOA4. Data are expressed as mean ± SD for 6 or 10 per group. Data were analyzed by parametric unpaired *t*-test. Values with asterisks (*) represent significant differences relative to the saline-treated group (*p* < 0.05).

Carnitine palmitoyltransferase (CPT) serves as a key regulatory enzyme of hepatic fatty acid oxidation in the mitochondria [38,39]. CD36 is a key scavenger receptor that promotes the transfer of long-chain fatty acid into liver cells [40]. To investigate whether or not APOA4 infusion stimulated hepatic sympathetic activity and regulated fatty acid oxidation and uptake in the liver, the NE turnover rate, and CPT1, CPT2 and CD36 levels in the liver were measured. Hepatic sympathetic activity and CPT2 levels in APOA4-treated mice were greater than those in saline-treated mice (*p* < 0.05, Figure 5A,B). In contrast, CPT1 levels in the liver were comparable between the APOA4 and saline treatment (Figure 5C). APOA4 infusion did not alter CD36 in the liver compared to that in the liver of saline-treated mice (Figure 5D), suggesting that APOA4 infusion did not change CD36-mediated lipid uptake by the liver. APOA4-treated mice had less TG and cholesterol in the liver than saline-treated mice had although they had comparable liver weights (*p* < 0.05, Figure 5E,F and Table 1). The findings suggest that APOA4 infusion may elevate hepatic sympathetic activity and attenuate hepatic lipids through the elevation of fatty acid oxidation but does not alter CD36-mediated fatty acid uptake by the liver.

**Figure 6:**
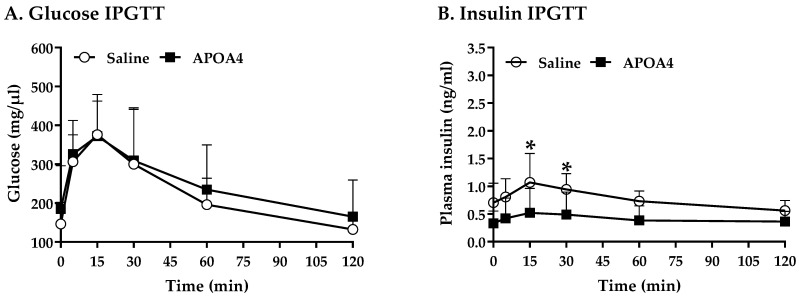
Serum glucose (**A**) and plasma insulin (**B**) over 120 min in response to an intraperitoneal glucose tolerance test. Data are expressed as mean ± SD for 8 or 10 mice per group. Values with asterisks (*) represent significant differences relative to the saline-treated group (*p* < 0.05).

BAT activation promotes whole-body glucose homeostasis [41,42]. Acute administration of APOA4 enhances glucose uptake by adipose tissues and improves glucose tolerance [21,43]. Figure 5 shows that APOA4 infusion attenuated hepatic triglycerides, suggesting that APOA4 infusion reduced hepatic glucose production. We investigated if APOA4 infusion could regulate whole-body glucose homeostasis. Prior to the intraperitoneal glucose tolerance test, basal glucose and insulin were similar in the APOA4- and saline-treated mice (Figure 6A,B). During the intraperitoneal glucose tolerance test, APOA4-treated mice had similar plasma glucose curves throughout the 120 min period (Figure 6A) but appeared to have a significant reduction in plasma insulin levels at 15 and 30 min after glucose challenge compared to the saline-treated mice (*p* < 0.05, Figure 6B). The findings suggest that APOA4 infusion may increase whole-body insulin sensitivity, accounting for normal intraperitoneal glucose tolerance in the presence of reduced glucose-stimulated insulin secretion.

## 4. Discussion

Dietary lipids elevate APOA4 levels and UCP1-dependent BAT thermogenesis [2,17,43,44]. BAT is highly innervated with sympathetic nerves [6]. Stimulation of sympathetic neurons induces NE release at nerve terminals [6,45]. Subsequently, the β3 adrenergic receptor signaling pathway induced by NE activates intracellular lipolysis and UCP1-dependent BAT thermogenesis [6]. In response to dietary lipids, APOA4-KO mice have decreased levels of NE synthesis and impaired UCP1-dependent BAT thermogenesis [24]. In contrast, acute administration of APOA4 enhances thermogenesis, sympathetic activity and fatty acid uptake in the BAT of chow-fed mice in response to dietary lipids [20]. The physiological role of continuous infusion of recombinant APOA4 protein in regulating sympathetic activity, thermogenesis, and lipid and glucose metabolism in LFD-fed mice remained elusive. The present experiments tested the hypothesis that a continuous infusion of APOA4 with a subsequent rise in plasma APOA4 would increase thermogenesis and consequently regulate lipid and glucose metabolism in LFD-fed mice. Because APOA4 secretion is induced by increased fat content [15], a LFD (5% caloric fat content) in the current experiment was used for minimizing endogenous lipid-induced APOA4 production by the small intestine in comparison to that with chow diets (14% caloric fat content) used in our previous reports [15,20]. This series of experiments demonstrated that male mice with increased levels of plasma APOA4, correlating with increased UCP1 and temperature in the BAT and contributing to enhanced BAT thermogenesis, improved hepatic lipid levels and glucose homeostasis, as well as decreasing plasma triglycerides in comparison with their saline control group, all without altering caloric intake and body weight. The effect of APOA4 in female mice remains to be tested due to sex-dependent BAT function [46].

Dietary lipids stimulate the sympathetic nervous system through the activation of vagal afferents for the elevation of sympathetic activity in adipose tissues and liver [2,47]. APOA4 acts on vagus nerves to relay neural signals to the hindbrain [48] that projects into the hypothalamus [47]. APOA4 has been reported to induce the neural activity of pro-opiomelanocortin neurons in the hypothalamic arcuate nucleus that further modulate downstream sympathetic activity [49,50]. The current experiments demonstrated that the induction of plasma APOA4 levels via APOA4 infusion elevated sympathetic activity, intracellular lipolysis and BAT thermogenesis. Thus, the current experiments suggest that the induction of BAT thermogenesis via APOA4 infusion resulted from a peripheral effect of APOA4. IWAT contains brown adipocytes with thermogenic capability [51]. Consistently with previous findings in APOA4-KO or transgenic mice with an overexpression of mouse APOA4 in the small intestine (APOA4-Tg mice) when maintained on a chow diet [23,24], the present experiments demonstrated that APOA4 infusion did not alter IWAT mass. In addition, APOA4 infusion was not able to enhance UCP1 or ATGL levels in IWAT through the induction of sympathetic activity. Our previous report [20] and the present study demonstrated that acute administration or continuous infusion of APOA4 elevated sympathetic activity and thermogenesis in the BAT and liver but not in the IWAT. The findings suggest that the action of APOA4 infusion on stimulation of sympathetic activity and thermogenesis differs between BAT and IWAT. 

Whole-body energy expenditure in lean APOA4-KO mice or lean APOA4-Tg mice was comparable to that of their wildtype controls during a standard diet feeding [23,24]. The current experiments showed that APOA4 infusion did not raise whole-body energy expenditure in lean mice during LFD feeding although it increased BAT thermogenesis and did not enhance IWAT thermogenesis. Consistently with previous findings [22,23,24,52], the present study indicated that APOA4 infusion did not reduce daily caloric intake in the LFD-fed mice. When fed a standard diet, APOA4-KO or APOA4-Tg mice and their controls utilize the same energy substrates for heat production [23,24]. The current study demonstrated that the RER in APOA4-treated mice was comparable to that in their saline-treated controls, and carbohydrate was used as the energy substrate for heat production. APOA4-KO or APOA4-Tg mice have comparable locomotor activity and body weight to those of their controls during chow feeding [23,24]. In the current experiments, APOA4 infusion did not alter locomotor activity, body weight or body composition during LFD feeding. These findings suggest that increased levels of plasma APOA4 resulting from APOA4 infusion may elevate UCP1-mediated BAT thermogenesis but do not raise whole-body energy expenditure and locomotor activity, and do not change caloric intake, the energy substrates required for heat production, and body weight or composition.

The sympathetic nervous system acts on BAT and IWAT (brown-in-white) adipocytes to stimulate fatty acid combustion and the liver for very low density lipoprotein (VLDL)-TG production to regulate TG metabolism [47]. Increased BAT thermogenesis attenuates hypertriglyceridemia through the induction of fatty acid combustion and TG clearance, and through fatty acid uptake by BAT providing the required fuel for this increase [8,37,53,54]. Using an APOA4-KO mouse model, APOA4 has been reported to alter TG transport-related intestinal gene expression, enhance chylomicron clearance, improve proximal TG transport, and increase fatty acid uptake by adipose tissues [20,24,55,56,57,58]. Intraperitoneal injection of APOA4 protein to wildtype mice assists in the activation of lipoprotein lipase, promotes the hydrolysis of TG-rich chylomicrons or VLDL, and enhances fatty acid uptake by BAT [20,59,60,61]. Consistently with previous findings which showed chow-fed APOA4-Tg mice to have reduced plasma TG [23], the current experiment showed that APOA4 infusion attenuated levels of TG in the plasma. The findings suggest that APOA4 infusion may induce BAT sympathetic activity for energy combustion, promote the delivery of fatty acids in chylomicron or VLDL to adipose tissues and consequently reduce the level of TG in the circulation for transportation to the liver for lipid metabolism. Obesity induces hypertriglyceridemia and increases the risk of cardiovascular diseases [62]. Patients with hypertriglyceridemia or cardiovascular disease have reduced plasma levels of APOA4 [63,64]. Further investigation regarding whether or not APOA4 infusion would downregulate hypertriglyceridemia through enhanced fatty acid uptake by adipose tissues and reduced lipids in lipoproteins in the circulation in lean and obese mice is needed.

The liver plays an important role in whole-body energy homeostasis by controlling lipid and glucose metabolism [65]. Sympathetic neurons are predominantly detected under neural innervation within the liver [66]. Sympathetic activation induces fatty acid oxidation for energy combustion to attenuate lipid content [38,67]. The current study demonstrated that APOA4 infusion stimulated sympathetic activity and elevated fatty acid oxidation in the liver. Fatty acid oxidation mainly occurs in the mitochondria and CPT plays a rate-limiting role in fatty acid oxidation [68]. CPT1 on the outer mitochondrial membrane catalyzes the conversion of long-chain acyl-CoA and carnitine into long-chain acylcarnitine and CoA, whereas CPT2 on the inner membrane converts them back into carnitine and acyl-CoA for fatty acid oxidation [68]. Hepatic denervation attenuates CPT2 expression in the liver of chow-fed rats [38]. The current experiments demonstrated that APOA4-induced sympathetic activity may be associated with the elevation of CPT2-mediated fatty acid oxidation. Because hepatic denervation does not alter CPT1 levels [38], APOA4-induced sympathetic activity likely had no effect on hepatic CPT1 levels in the current experiments. Whether or not the direct effect of APOA4 on sympathetic activation could raise mitochondrial CPT2-induced fatty acid oxidation remains to be investigated. The current study demonstrated that APOA4 infusion did not attenuate CD36-mediated fatty acid uptake in the liver. Consistently with the reduction in hepatic TG content found in transgenic mice with an overexpression of APOA4 in the liver [69], the current experiment showed that APOA4-treated mice had lower hepatic TG content than saline-treated mice had, possibly due to increased CPT2-mediated fatty acid oxidation in the liver. Consistently with the findings in our previous report using transgenic mice that had an overexpression of mouse APOA4 in the small intestine [23], APOA4 infusion did not alter plasma cholesterol and leptin levels in the current study. 

Stimulation of BAT thermogenesis with enhanced hepatic fatty acid oxidation improves insulin resistance [67,70,71]. APOA4-KO mice have impaired glucose tolerance, glucose-induced insulin secretion, and insulin sensitivity, suggesting that APOA4 may improve glucose homeostasis and insulin sensitivity [21,72]. In contrast, APOA4 suppresses hepatic gluconeogenesis and glucose production, promotes glucose uptake by BAT and white adipose tissues and enhances insulin sensitivity [43,72,73]. In the current experiments, APOA4 infusion attenuated hepatic TG and plasma glucose-induced insulin levels to maintain glucose homeostasis in response to a glucose challenge, suggesting that APOA4 infusion may increase insulin sensitivity in LFD-fed mice. The findings suggest that APOA4 infusion attenuates hepatic TG content resulting from the elevation of sympathetic activity and fatty acid oxidation in the liver and does not reduce fatty acid uptake and cholesterol levels in the liver of LFD-fed mice. Additionally, APOA4 infusion improves insulin sensitivity in LFD-fed mice. 

## 5. Conclusions

Activating BAT thermogenesis has great potential in combating obesity, insulin resistance, and cardiovascular diseases [1,8,12,70]. The present study demonstrated that the physiological role of APOA4 infusion was to stimulate sympathetic activity, elevate UCP1-dependent BAT thermogenesis and fatty acid oxidation in the liver, and consequently attenuate plasma and hepatic TG and insulin levels without altering caloric intake, body weight gain and fat mass. The next step will be to investigate the ability of continuous APOA4 infusion to attenuate potential obesity-related complications through the elevation of BAT thermogenesis and downregulation of hypertriglyceridemia in the face of a HFD challenge. 

## Figures and Tables

**Table 1:** Body weight, fat mass, and levels of plasma parameters.

Parameters	Saline	APOA4
Body weight (g) before treatment	25.5 ± 1.91	25.5 ±1.85
Body weight (g) after treatment	27.9 ± 1.51	27.5 ± 1.79
Body weight gain (g)	3.0 ± 1.63	2.7 ± 0.84
Daily caloric intake (Kcal)	7.7 ± 1.47	7.5 ± 0.45
Fat mass/body weight (%)	12.9 ± 1.84	12.4 ± 1.37
Lean mass/body weight (%)	72.2 ± 2.01	72.5 ± 1.96
BAT (g)	0.14 ± 0.05	0.17 ± 0.06
IWAT (g)	0.31 ± 0.07	0.31 ± 0.09
EWAT (g)	0.34 ± 0.05	0.41 ± 0.08
Liver (g)	1.24 ± 0.16	1.14 ± 0.21
Triglycerides (mg/dL)	42.0 ± 11.05	29.0 ± 5.63 *
Cholesterol (mg/dL)	111.6 ± 21.01	120.7 ± 10.66
Leptin (ng/mL)	1.8 ± 0.72	2.1 ± 1.01
Insulin (ng/mL)	0.32 ± 0.29	0.33 ± 0.24

Body weight, caloric intake, lean mass, and fat mass were monitored when mice (n = 8 or 10 mice per group) were maintained on a LFD. Body weight, tissues, and plasma were collected after a 5 h fast. Daily caloric intake is the average of seven days of caloric intake measured during the 10th week of LFD feeding. Lean mass or fat mass was normalized to body weight. Values are represented as mean ± SD. Data were analyzed using a parametric unpaired *t*-test. Values with asterisks (*) represent significant differences relative to the saline-treated mice (*p* < 0.05).

## Data Availability

The data that support the findings of this study are available upon request from the corresponding author.

## Supplementary Materials

The following supporting information can be downloaded at https://www.mdpi.com/article/10.3390/nu15112486/s1. Figure S1: Acute or daily administration of APOA4 increases BAT thermogenesis.
