# Supplementary material for: Apolipoprotein A4 Elevates Sympathetic Activity and Thermogenesis in Male Mice
Source: Nutrients. 2023 May 26;15(11):2486. doi: 10.3390/nu15112486 (PMC10255745; doi:10.3390/nu15112486)
Supplement: Supplementary file 1 [file nutrients-15-02486-s001.zip › nutrients-2386652-supplementary.pdf]

Supplementary

Acute or daily administration of APOA4 increases BAT thermogenesis

To examine whether acute injection of APOA4 can regulate BAT thermogenesis, BAT thermogenic protein in chow-fed mice was determined after they received an acute intraperitoneal injection of APOA4 protein at different doses. Acute injection of APOA4 at 0.6 mg/kg or above significantly elevated BAT UCP1 protein relative to saline treatment ($p < 0.05$, Supplementary Figure S1A).

To investigate whether daily administration of APOA4 at 1.2 mg/kg can elevate BAT thermogenesis in chow-fed mice at normal ambient temperature, UCP1, a marker for BAT thermogenesis, was measured. Mice at 12 weeks of age were divided into two feeding groups with matched body weight (BW). The two groups of mice received a chow diet while housed at 21 ± 0.5°C for 3 weeks. During the 3 weeks, one group received intraperitoneal injections of saline and the other group received APOA4 at 0.6 mg/kg twice a day (total amount: 1.2 mg/kg/day), at 12:00 and 19:00. This dose of APOA4 was previously determined as the lowest effective dose for induction of BAT thermogenesis (Supplementary Figure S1A). On the last day of treatments, all mice were fasted for 5 hours and then their blood was collected using a blood collecting tube (Kimble #42E603, Rockwood, TN, USA) and microtainer (BD, #365967, Franklin Lakes, NJ, USA). The BAT of 5-hour fasted mice was collected on dry ice for further analysis. Daily administration of APOA4 at 1.2 mg/kg increased UCP1 proteins in BAT relative to their controls ($p < 0.05$, Supplementary Figure S1B). The findings suggest that acute injection of APOA4 at 0.6 mg/kg or daily administration of APOA4 at 0.6 mg/kg twice a day (1.2 mg/kg total) elevates UCP1-dependent BAT thermogenesis.

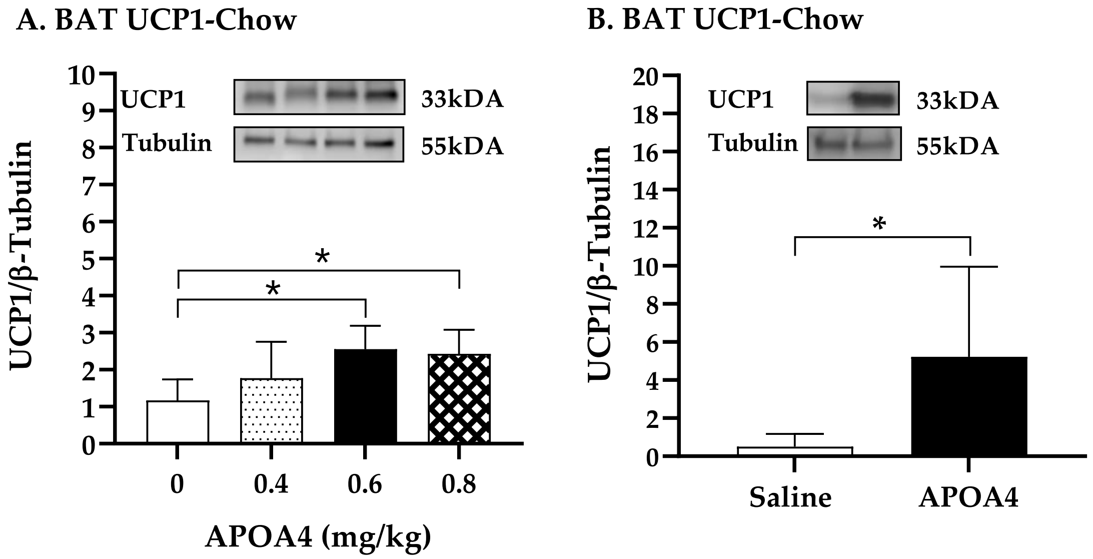

Supplementary Figure S1. Thermogenic protein in BAT of mice with an acute injection of APOA4 at different doses (A) and with daily administration of APOA4 at 0.6 mg/kg twice a day (B) when fed a chow diet for 3 weeks at 21°C. Data are expressed as mean ± SD for 5-6 animals per group.
